# The Sensor Modules of a Dedicated Automatic Inspection System for Screening Smoked Sausage Coloration

**DOI:** 10.3390/s26020678

**Published:** 2026-01-20

**Authors:** Yen-Hsiang Wang, Yu-Fen Yen, Kuan-Chieh Lee, Ching-Yuan Chang, Chin-Cheng Wu, Meng-Jen Tsai, Jen-Jie Chieh

**Affiliations:** 1 Institute of Electro-Optical Engineering, National Taiwan Normal University, Taipei 116, Taiwan; 80648001s@ntnu.edu.tw (Y.-H.W.); 81277001h@ntnu.edu.tw80648003s@ntnu.edu.tw (K.-C.L.); 81377002h@ntnu.edu.tw (C.-Y.C.); 2 Product and Process Research Center, Food Industry Research and Development Institute, Hsinchu 300, Taiwan; mjt@firdi.org.tw; 3 Department of Biomechatronic Engineering, National ILAN University, Yilan City 260, Taiwan; chincheng@niu.edu.tw

**Keywords:** handheld colorimeter, in-line automated sorter, color classification, food quality control, CIE LAB color space

## Abstract

The external color of smoked sausages is a critical indicator of quality and uniformity in processing. Commercial colorimeters are unsuitable for high-throughput sorting due to the challenges posed by the sausage’s curved cylindrical surface and the need for an inline application. This study introduces a novel non-contact sensing module (LEDs at 45°, fiber optic collection at 0°) to acquire spectral data (400–700 nm) and derive CIE LAB. First, a handheld prototype validated the accuracy of the sensing module against a benchtop spectrophotometer. It successfully categorized five color grades (‘Over light’, ‘Light’, ‘Standard’, ‘Dark’, and ‘Over dark’) with a clear distribution on the a*-L* diagram. This established acceptable color boundary conditions (44.2 < L* ≤ 61.3, 14.1 < a* < 23.9). Second, three sensing modules were integrated around a conveyor belt at 120° intervals, forming the core of an automated inline sorting system. Blind field tests (*n* = 150) achieved high sorting accuracies of 95.3–97.3% with an efficient inspection time of less than 2 s per sausage. This work realizes the standardization, digitalization, and automation of food color inspection, demonstrating strong potential for smart manufacturing in the processed meat industry.

## 1. Introduction

Sausages are popular processed meat products, typically prepared from pork, chicken, or beef, combined with spices, seasonings, and food additives. Their production involves several essential steps, including comminution or homogenization, curing, stuffing, and drying [1]. The smoke-curing process is particularly valued. It produces unique flavors and characteristic colors, making smoked sausages highly favored by consumers [2,3]. The external color of smoked sausages is not merely a superficial trait; it is a direct proxy for the extent of the smoke-curing process and is critically viewed as a visual representation of product quality, flavor, and freshness. Consequently, it serves as a major quality control metric [2,4]. However, in large-scale food manufacturing (with an annual output greater than 3000 tons), several factors often lead to significant color discrepancies within the same batch. These include variability in raw material specifications (e.g., variations in the lean-to-fat ratio and surface moisture content) and non-uniformity in the heating or smoking apparatus, even under identical processing conditions. This non-uniformity results in frequent customer complaints, product returns, and subsequent financial losses for the industry. Currently, quality control (QC) in most food processing plants relies heavily on manual inspection, where trained personnel visually assess the product’s external color for sorting. This method is labor-intensive and creates a significant bottleneck in production, as a single trained QC specialist is typically limited to inspecting approximately 150 samples per day. Furthermore, human sensory evaluation is inherently subjective and prone to misjudgment, undermining the goal of product standardization. Therefore, the development of rapid, non-destructive, and inline applicable sensor technology for the objective analysis of a product’s external color is highly imperative. Such an innovation would significantly contribute to the standardization of product quality, effectively boosting the digitalization of the food industry and facilitating the transition toward automation and smart manufacturing practices.

Scientific analysis of food color quality is widely based on colorimeters [4,5]. Benchtop spectrophotometers offer high accuracy and stability, making them ideal for laboratory analysis of a wide range of samples. However, their large footprint and fixed operating environment make them unsuitable for real-time inspection on a dynamic production line [5]. Handheld colorimeters possess the advantages of high portability and the ability to capture color values instantly on-site or near the production line. Nonetheless, their accuracy is susceptible to ambient light conditions and operator handling [5]. Crucially, neither of these traditional methods can be seamlessly integrated into automated systems, limiting their use in large-scale production and automated quality management.

Automated Optical Inspection (AOI) utilizes high-resolution imaging and machine vision technology to perform mass, automated image inspection, providing real-time data feedback. This significantly reduces human visual inspection errors and operating costs [6,7,8]. Its advantages include non-contact, comprehensive, and high-speed inspection, making it particularly effective for precise surface defect detection on large, continuous materials [6]. While AOI is predominantly applied and has been proven effective in industries like printing, optics, and electronics [6,7,8,9], its primary focus is often on analyzing surface morphological differences rather than comprehensive chromaticity. Furthermore, AOI systems demand a stringent optical design and involve high capital expenditure, making their investment challenging for the food industry, which is often price-sensitive.

Color Space Application Spectrophotometry, which measures the complete visible light spectrum (400–700 nm), is the standard tool for quality control of color in food, printing, and cultural heritage. It can detect minimal color differences (ΔE < 1) and offers absolute traceability [5,10]. Numerous studies have demonstrated the feasibility of using visible colorimetric systems to monitor food quality. Spectral data (340–750 nm) converted to RGB/CIE LAB color space (CIE LAB) has been used for non-destructive spoilage detection in sliced bread, achieving an F1 score of 95.84% in Support Vector Machine (SVM) testing for shelf-life evaluation [10]. The CIE LAB was employed to establish a color kinetic model for pine bark under thermal treatment (80–200 °C), with the coefficient of determination (R^2^) consistently exceeding 0.9782, confirming its predictive reliability [11]. Research also indicates a significant correlation between the oxidation indicators of coriander seed oil and the ΔE value in CIE LAB, positioning it as a viable basis for non-destructive quality assessment [12]. Using Hunter LAB color space in combination with algorithms has achieved grading accuracy for seed cotton with R^2^ ranging from 0.809 to 0.879 [13]. Furthermore, the CIE LAB, coupled with Random Forest (RF) regression, successfully predicted the spoilage rate of beef, yielding an outstanding R^2^ of 0.988 and a root mean square error (RMSE) of 2.120 [14].

Based on the limitations of conventional colorimeters and the high cost/specificity of AOI, coupled with the proven efficacy of spectrophotometry and CIE LAB in food quality assessment, there is a clear necessity for an accurate, cost-effective, and production-line-ready sensing solution. Therefore, this study proposes the development of an automated inspection system module that utilizes visible-reflected spectral data converted to CIE LAB. This system is designed to objectively classify the color of smoked sausages into five grades of smoking intensity: ‘Over light’, ‘Light’, ‘Standard’, ‘Dark’, and ‘Over dark’. Through modular design and rigorous blind-test validation, the goal is to provide food manufacturers with a highly accurate and stable operable color sensor. This system implements visual grouping and classification rules to achieve automated sample grading and anomaly detection, thereby implementing scientific and real-time process control for food color on the production line. This increases efficiency and advances automated development.

## 2. Materials and Methods

### 2.1. Design of Development Processes

**Figure 1:**
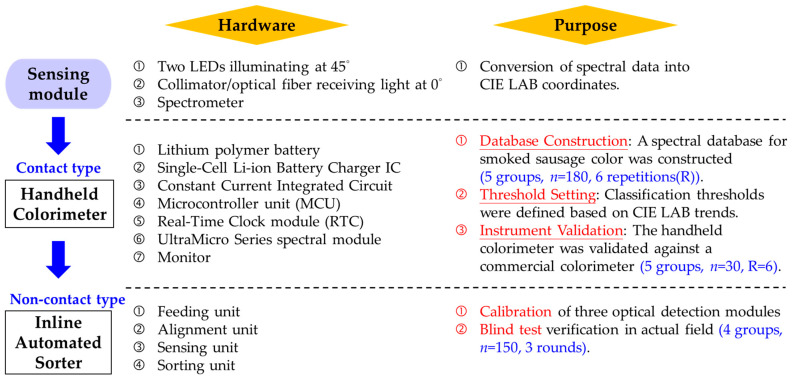
Overall experimental design.

The entire study was conducted in two distinct phases, as schematically illustrated in Figure 1.

Phase I: Handheld Colorimeter Development and Sensor Validation

The primary objective of Phase I was to develop a handheld colorimeter prototype to validate the proposed sensing module technology. This phase encompassed the following critical steps. Spectral Data Conversion: A robust conversion algorithm was established from raw spectral data (400–700 nm) and converted to the CIE LAB. Threshold Setting: The classification thresholds were defined for different degrees of smoked sausage color. The color categories of the product samples (5 groups, *n* = 180) were determined objectively by consensus voting among four experienced quality control professionals (trained panelists) from the collaborating food company, based on visual assessment. Instrument Validation: The colorimetric values obtained by the newly developed sensing module were rigorously compared against results from a commercial benchtop spectrophotometer to confirm its accuracy and reliability. All color analyses were performed in six replicates to ensure statistical robustness.

Phase II: Automated Inline Sorting System Implementation

Phase II focused on scaling up the validated technology into a production-ready system. This involved utilizing three identical sensing modules, strategically placed to enable multi-directional sensing of the sausage surface. These modules were integrated into a newly developed automated Inline sorting machine on the production line. A comprehensive three-round blind field test was conducted, using *n* = 150 samples per round, to verify the system’s accuracy, stability, and operational performance under industrial conditions.

### 2.2. Sample Classification for Color Database

**Figure 2:**
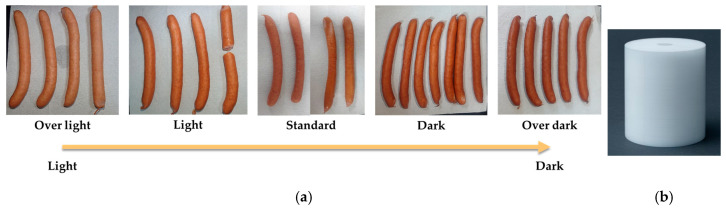
(**a**) The five categorized color grades of smoked sausages: ‘Over light’, ‘Light’, ‘Standard’, ‘Dark’, and ‘Over dark’. (**b**) The calibration standard used for the white reference measurement.

The samples used in this study consisted of *n* = 180 smoked sausages, sourced from a collaborating food processing plant in Pingtung County, Taiwan. As the largest and longest-established livestock processing enterprise in Taiwan, this facility produces a diverse portfolio of over 300 product types, encompassing fresh pork cuts and processed meat products such as hams, meat floss, jerky, and prepared foods. The company maintains a total annual yield exceeding 18,000 tons; notably, the annual production of smoked sausages alone surpasses 3000 tons. The smoked sausages selected for this study represent typical ‘original flavor’ products manufactured using standard industrial protocols, with no intentional deviations in the preparation method. These samples were approximately 16–18 cm in length. For the purpose of establishing the color database, these samples were initially categorized into five distinct color grades based on consensus visual assessment and voting by four trained QC specialists from the facility. The five color grades, representing the degree of smoking/thermal processing, are defined as ‘Over light’, ‘Light’, ‘Standard’, ‘Dark’, and ‘Over dark’. An example illustrating these five categories is presented in Figure 2a.

### 2.3. Preparation of the White Calibration Standard

Polytetrafluoroethylene (PTFE) was selected as the reference reflection standard material (Figure 2b) for spectrophotometer calibration in this study. This choice was primarily based on the following four advantageous characteristics. High Physico-Chemical Stability: PTFE exhibits excellent chemical inertness and thermal stability, ensuring its physical and optical properties are maintained even under harsh conditions such as high temperatures or humidity. This makes it suitable as a long-term calibration reference. Ease of Cleaning and Maintenance: Due to its hydrophobic and non-adhesive surface characteristics, PTFE does not easily absorb moisture or contaminants when used. It can be readily cleaned via dry or wet wiping, resulting in low maintenance costs. High and Stable Reflectance: Sintered white PTFE generally exhibits a high total reflectance exceeding 90% across the ultraviolet to visible range (250–800 nm). Crucially, it demonstrates near-Lambertian reflectance, offering excellent uniformity and diffuseness. Proven Practical Application: White PTFE has demonstrated its consistency and repeatability when applied as a reference standard in the calibration of hyperspectral imaging systems [15,16]. Furthermore, the surface of the PTFE standard was subjected to mechanical sanding using 800- to 1000-grit aluminum oxide sandpaper. This process was performed to achieve multiple objectives [17]: to remove surface contamination and any slight oxidation layers, to create a uniform micro-surface roughness structure, to minimize specular reflection (mirror-like) and enhance diffuse reflectance distribution characteristics, and to maintain its inherent non-conductivity and chemical resistance.

### 2.4. Conversion from Spectral Data to CIE LAB

The spectrometer (UM2280, OtO Photonics, Hsinchu, Taiwan) classifies color data based on electromagnetic wavelength with a resolution of 1 nm. Conversely, the CIE LAB (L*, a*, b*) is an internationally recognized model designed to simulate human visual perception (lightness, red–green axis, and yellow–blue axis). Therefore, a standardized conversion procedure is required to transform the measured spectral information into color values that correlate with human vision. This algorithmic conversion is widely applied in color science and industries requiring high color consistency, such as food, textiles, coatings, and plastics [4,5,18,19].

Unlike RGB-based imaging sensors, which require device- and illumination-dependent color calibration, the proposed system employs a full-range visible spectral module to enable traceable colorimetric computation based on standard illuminants and observers. By varying levels of illumination, this method enhances measurement robustness, which is critical for accurate food quality and safety inspection.

Step 1: Data Preparation
  - Spectral Reflectance R(λ): Sampling was carried out every 5 nm from 400 nm to 700 nm, resulting in 61 data points.
  - CIE 1931 2° Standard Observer Color-Matching Functions [20]: $\bar{x}$(λ), $\bar{y}$(λ), and $\bar{z}$(λ) mathematically describe the sensitivity of the human eye to different wavelengths.
  - Standard Illuminant Spectral Power Distribution S(λ): This refers to the power distribution of the chosen standard light source (e.g., D65 daylight).
Step 2: Calculation of Tristimulus Values (XYZ)

The Tristimulus values (X, Y, Z) are calculated using the following integration formulas (typically via numerical integration or matrix multiplication):

(1) $$X=k\sum R\left(\lambda \right)S\left(\lambda \right)\bar{x}(\lambda )\;Y=k\sum R\left(\lambda \right)S\left(\lambda \right)\bar{y}\left(\lambda \right)\;Z=k\sum R\left(\lambda \right)S\left(\lambda \right)\bar{z}(\lambda )$$

where k is the normalization constant defined as Y = 100 for the perfect reflecting diffuser under the specified illuminant (i.e., R(λ) = 1):

(2) $$k=\frac{100}{\sum S\left(\lambda \right)\bar{y}(\lambda )}$$

Step 3: Conversion from XYZ to CIE LAB

The calculated (X, Y, Z) values are then converted to (L*, a*, b*) by referencing the Tristimulus values of the reference white point (X_n_, Y_n_, Z_n_) under the specified D65 illuminant. First, the function f(t) is defined as follows:

(3) ft= t1/313(296)2t+429  , if t>(629)3 , if t≦(629)3 

The CIE LAB coordinates are then calculated:

(4) $$L^{*}=\;116f\left(\frac{Y}{Y_{n}}\right)-16\;a^{*}=\;500\left[f\left(\frac{X}{X_{n}}\right)-f(\frac{Y}{Y_{n}})\right]\;b^{*}=\;200\left[f\left(\frac{Y}{Y_{n}}\right)-f(\frac{Z}{Z_{n}})\right]$$

CIE XYZ and CIE LAB values were computed from measured spectral data following ASTM E308, which enables compatibility with standard colorimetric definitions rather than device-specific RGB mappings [21,22].

### 2.5. Sensing Module and Handheld Colorimeter

#### 2.5.1. Mechanical Design

The sensing module adopts the CIE standard 45°/0° measurement geometry, which is crucial for effectively enhancing the signal-to-noise ratio (SNR) and overall measurement stability. Dual white light-emitting diodes (LEDs) (LTW-2R3D7, Lite-On Inc., Taipei, Taiwan) are symmetrically configured at a narrow 45° angle on both sides. This setup simulates human viewing conditions, provides uniform illumination, and prevents stray light from reaching the collector. The light collection module is positioned in the 0° direction. It first collects vertically reflected light via a collimating lens (COL-1-UV, OtO Photonic, Taiwan). This light is then transmitted to the spectrometer using an optical fiber (OF-DS-1000-A, OtO Photonic, Taiwan) with a numerical aperture (NA) of 0.22 and a corresponding acceptance angle of ±12.7°. The two optical components of the collection module are precisely matched to optimize coupling efficiency. The main body of the device is constructed from black Poly Lactic Acid (PLA) and Polyethylene Terephthalate Glycol (PETG), coated with flat black paint to suppress unwanted internal reflections. The optical path mechanism incorporates threaded holes and slot structures, supplemented by chamfer and groove positioning designs to ensure precise alignment of the optical center. A critical feature is the disposable sample contact surface made of food-grade polypropylene (PP). This design provides good mechanical strength and chemical resistance, is detachable for cleaning to ensure sanitation, and thus maintains low maintenance costs. Furthermore, the interior features a curved surface precisely designed to conform to the cylindrical shape of the sausage samples, thereby enabling consistent contact and measurement reproducibility.

**Figure 3:**
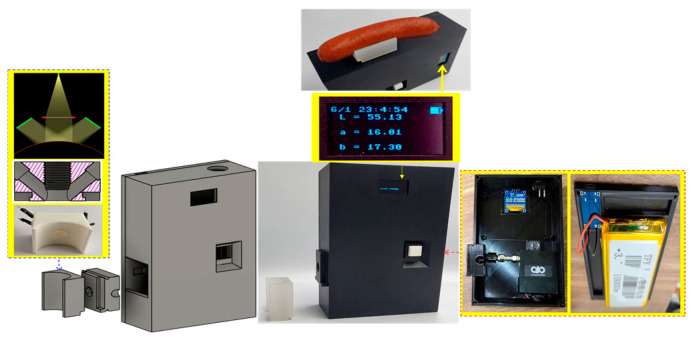
Handheld colorimeter: its appearance and internal diagram.

To enable rapid on-site data acquisition for color database construction, the sensing module was integrated with essential electronic components to construct a handheld colorimeter (Figure 3). These components included a microcontroller (ESP32-WROOM-32, Espressif Systems Co., Ltd., Shanghai, China), a real-time clock (DS1307, Analog Devices, Inc., Wilmington, MA, USA), a display (SSD1306, Solomon Systech Limited, Shanghai, China), and a battery (1165110, Shenzhen Sunhe Energy Co., Ltd., Shenzhen, China). The operating buttons were ergonomically arranged for single-handed operation and included an independent power switch, which collectively improved operational convenience and safety in the field. The detailed specifications and functions of the handheld colorimeter’s components are listed in Table 1.

#### 2.5.2. Spectrometer and Light Source Selection

The selection of the light source was critical due to the non-uniform sensitivity of the spectrometer used. Specifically, the spectrometer exhibits relatively low sensitivity in the blue light region (400–500 nm) but shows a high response in the red light region (650–750 nm), particularly around 680–700 nm [23] (Figure S1a).To compensate for this spectral imbalance, selecting a white LED with high output in the red region, such as the 334-15/X1C2-1UWA/EU (Everlight Electronics Co., Ltd., New Taipei City, Taiwan) [24], would result in a red-shifted spectrum detected by the spectrometer. Therefore, it was necessary to select an LED specification with a relatively strong blue light output. Two white LEDs, the C512A-WNS/WNN (Cree Inc., Durham, NC, USA) and the LTW-2R3D7, both exhibited a prominent peak in the blue light band (around 450 nm) [25]. The LTW-2R3D7 was ultimately selected for the following reasons. Balanced Output: It provides a sufficiently high output in the blue light band to compensate for the spectrometer’s low sensitivity. Stable Intensity: Its intensity is moderate across the yellow-to-red light bands, which helps maintain average and stable light intensity across the spectrum [26]. Optimal Beam Angle: Its maximum emission angle of 35° closely matches the design requirement of the sensing module, facilitating the desired 45° illumination geometry (Figure S1b).

#### 2.5.3. Light Source Driving and Control Methodology

To guarantee the stability and reproducibility of the light source intensity, this study employed a constant current (CC) driving method, replacing the conventional constant voltage supply. This approach effectively prevents fluctuations in light output and potential spectral shifts caused by voltage or load variations, thereby ensuring the accuracy of the colorimetric data. A low-cost, stable current circuit was implemented using a linear current regulator (LM317, TI, USA) (Figure S2a). With a 5 V input and a setting resistance R_1_ of 62Ω, the circuit yielded an output current of approximately 20.2 mA. This linear CC method is characterized by low noise, minimal ripples, and high anti-interference capability. Compared to Pulse Width Modulation (PWM) or switching regulators, it features simpler components, linear response, and lower cost, making it ideal for the medium-to-low current requirements of LED-based color measurement systems. To confirm that the linear CC circuit prevents light degradation (lumen depreciation) caused by rising temperatures during prolonged operation, the system’s stability was validated through two critical parameter measurements:

(1) Current Stability: The current was continuously monitored for 10 min at 25 °C using a digital multimeter (Prokit, MT-1820, London, UK). The resulting current variation rate was merely 0.50%, which is significantly lower compared to the 3.73% variation rate observed without the CC regulation (Figure S3).
(2) Light Intensity Stability (Luminosity): Measurements were taken inside a dark box using the spectrometer, sampled every second for 10 min. The maximum light intensity fluctuation was approximately 0.58%, which significantly outperformed the 5.52% fluctuation rate recorded without the constant current drive (Table S1).

In conclusion, the constant current driving method effectively suppressed voltage and current drift. This resulted in a significant improvement in LED brightness stability and measurement reproducibility, thereby ensuring the accuracy and reliability of the subsequent spectral and color difference analyses.

#### 2.5.4. Electronic Circuit Design and Operation Procedure

**Figure 4:**
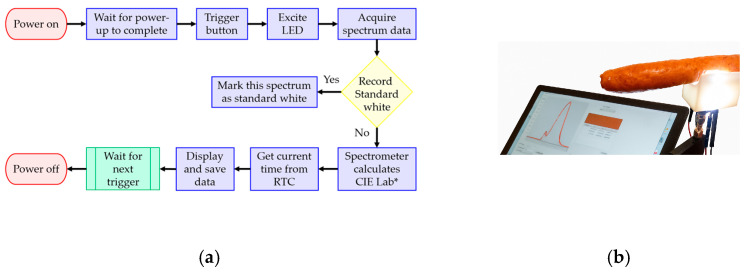
Color measurement process for sausages: (**a**) electronic operation flow; (**b**) measured spectrum and calculated L*, a*, b*.

The overall system architecture and circuit diagram for the handheld colorimeter are detailed in Figure S2. The system integrates several key components: ESP32 control logic (Figure S2b), a lithium battery power supply (Figure S2c), the TP4056 charging management module (Figure S2d), the DS1307 real-time clock (RTC) (Figure S2e), and the SSD1306 display module (Figure S2f). The ESP32 control logic serves as the central processing unit, managing the spectrometer, power configuration, and OLED display. It performs real-time calculation of colorimetric values, handles data display, and manages storage functions. The system also supports connection to a personal computer for advanced analysis, including the visualization of the measured spectrum (Figure 4b). The overall system operation procedure (Figure 4a) takes the form of the following sequence: Device Power-Up→Execution of White Standard Measurement (Calibration)→User Triggered Measurement (Button Press)→Activation of LED Illumination→Reflected Light Acquisition→Conversion to CIE LAB Values→Display and Storage of Measurement Results.

The measurement results are displayed instantly on the device screen (Figure 4b). Future expansions may include wireless transmission and cloud synchronization via an optional module. Consequently, users can complete the sample color analysis by simply pressing the operation button with a single hand. The results are returned to the screen within 2 s, demonstrating an intuitive, rapid, and user-friendly operation highly suitable for on-site quality control applications.

#### 2.5.5. Modeling, Validation, and Establishment of Color Grading Thresholds

A color difference database was established using the handheld colorimeter to measure the CIE LAB of 180 smoked sausage samples (*n* = 180) across five color categories previously defined by the four trained QC specialists (*n* = 4). To ensure the fidelity of this newly constructed database, a subset of 30 representative samples (*n* = 30)—selected to cover all five smoking intensity grades—was measured using a commercial benchtop spectrophotometer (CM-5, Konica Minolta Inc., Tokyo, Japan). Each sample was measured in six replicates. Since the commercial spectrophotometer did not provide raw spectral data, the validation focused on comparing the trend consistency of L*, a*, and b* between the two instruments. Absolute numerical differences were expected due to variations in optical hardware, software settings, and measurement geometries/spot sizes.

Following the confirmation of the database’s fidelity, the next step was to define the upper and lower boundary thresholds (acceptance criteria) based on the colorimetric values that significantly influence classification. The procedure involved the following steps. (1) Statistical Analysis: The maximum, minimum, and quartiles were calculated for L*, a*, and b* across all samples within each of the five groups (‘Over light,’ ‘Light,’ ‘Standard,’ ‘Dark,’ ‘Over dark’). (2) Boundary Definition: These statistical parameters were utilized to establish distinct boundary ranges that serve as the classification thresholds for different smoking color grades. This process of establishing classification standards is vital, as it digitally models and replaces the subjective classification performed by skilled personnel, enabling subsequent high-throughput and rapid sorting on the production line. The final defined classification thresholds for acceptance were 44.2 < L* ≤ 61.3 and 14.1 < a* < 23.9. These thresholds successfully partition the samples into five color grades based on smoking intensity.

### 2.6. Development of the Color Sensing Module for the Inline Automated Sorting Machine

**Figure 5:**
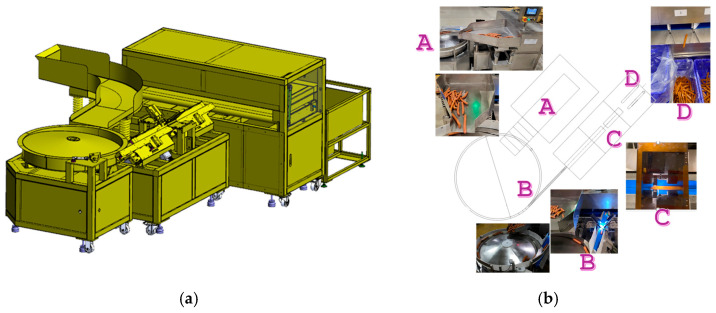
(**a**) External view of the inline automated sorting machine. (**b**) Schematic diagram illustrating the four functional unit zones of the machine.

The handheld colorimeter prototype primarily served to validate the sensing module and the data-to-classification algorithm. To meet the requirements of high-volume, piece-by-piece product sorting, the inline automated sorting machine was used (Figure 5).

#### 2.6.1. Inline Automated Sorting Machine

The sorting machine comprises four functional units, detailed in Table 2. The Feeding Area (Unit A) features a gentle slope to allow batches of smoked sausages to flow down gradually. The Alignment Area (Unit B) contains ring-shaped grooves that accommodate single sausages, serving to filter out excessively curved samples. The Optical Inspection Area (Unit C) is where the smoked color is assessed. The Sorting Area (Unit D) directs the classified sausages into different collection bins based on the determined color grade (Figure 5b). The Alignment module (TM-A010, Yi Sun Automation Co., Ltd., Taichung City, Taiwan) is responsible for positioning the sausages in a single file. The Sorting module (TM-A012, Yi Sun Automation Co., Ltd., Taiwan) is a four-lane sorter. The sensing region lies between the Alignment and Sorting modules.

**Figure 6:**
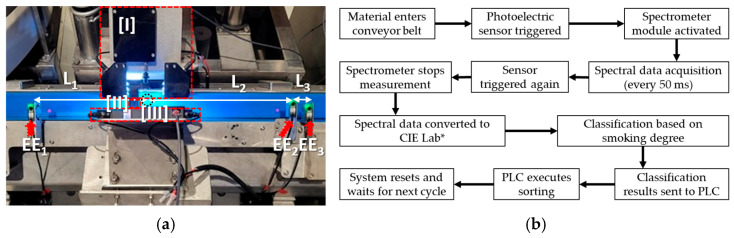
Inline automated sorting machine: (**a**) installation layout ([I], [II], and [III] indicate the positions of the three sensors. EE1, EE2, and EE3 indicate the positions of the three photoelectric sensors). (**b**) Workflow diagram.

Three photoelectric sensors (EE1, EE2, EE3, SICK, G6, Waldkirch, Germany) are installed before and after the sensing region to serve as triggering conditions (Figure 6a). These sensors calculate or monitor the length and position of the sausages on the V-structure conveyor belt, thereby controlling the components in the Optical Inspection and Sorting Areas (Figure 6b). The automated sorting procedure is outlined as follows. (1) Entry and Trigger: A sample enters the conveyor belt, and the photoelectric sensor EE1 triggers the start signal. (2) Measurement Initiation: The sensing module’s spectrometer initiates measurement of the sausage’s reflected spectrum, acquiring data every 50 milliseconds until sensor EE2 triggers the end-of-measurement signal. Classification and Sorting: (3) The spectral data are converted to CIE LAB. Based on the classification thresholds, the programmable logic controller (PLC) activates the sorting module actions. (4) Process Reset: Sensor EE3 triggers a signal to clear the process, preparing the system for the next sample. This comprehensive procedure achieves fully automated alignment, color sensing, and classification of smoked sausages on the production line.

#### 2.6.2. Optical Inspection Unit

**Figure 7:**
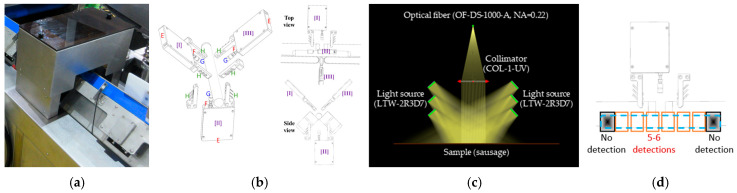
The Optical Inspection Unit for the inline automated sorting machine: (**a**) appearance; (**b**) 3D detection configuration of three sensing modules; (**c**) the illumination and detection configuration of one sensing module; (**d**) the precision segmentation of one sausage into 5–6 measurement regions.

The Optical Inspection Unit (Figure 7a) comprises three identical sensing modules (Figure 7b), strategically positioned around the cylindrical sample on the conveyor belt, spaced every 120°. Unlike the contact-based handheld colorimeter, the Optical Inspection Unit is non-contact. Due to the distance between the collecting fiber’s front lens and the sample (45 mm), the illumination system was modified to include three pairs of light sources (Figure 7c). The distances from these three pairs of light sources to the sample are set to 54 mm, 48 mm, and 42 mm, respectively. Each of the three sensing modules utilizes a configuration where the light source is incident at 45° (from two sides) and the light collection fiber is positioned at 0° (central reflection), strictly adhering to the CIE standard color measurement conditions.

The system utilizes photoelectric sensors (electric eyes), time tracking, and conveyor belt speed calculations to precisely monitor the measurement position range on the sausage. Considering the sausage length is approximately 16–18 cm, the first 2 cm of the leading end is excluded from the measurement. Given the conveyor belt speed of 0.45 m/s and a maximum spectral integration time of 50 ms per measurement cycle for the three modules, each sausage generates 5 to 6 measurement zones (Figure 7d), yielding a total of 15–18 reflected spectral data points across a sensing length of approximately 12–14 cm. The spectral data from the three sensing modules are converted into CIE LAB using the established CIE 1976 standard algorithm. L*, a*, and b* are then averaged to represent the final color assessment of a specific location on the smoked sausage. Summarily, the Optical Inspection Unit is a multi-point measurement system; three spectrometer modules (Module [I]–[III]) were integrated as three independent channels mounted at different positions to enable simultaneous acquisition from multiple locations on the sample.

#### 2.6.3. Field Blind Test Validation

To validate the classification performance and operational stability of the developed inline automated sorting machine, a comprehensive blind field test was executed. Given the limited daily inspection capacity of the QC specialists, the sample size for the blind test was standardized at 150 sausages. The process began with four trained QC specialists (*n* = 4) randomly selecting smoked sausages from the production line to serve as blind test samples. The ground truth (reference classification) was established based on the trained QC specialist’s visual assessment: a consensus agreement classifying the sample as ‘Light,’ ‘Standard,’ or ‘Dark’ defined it as an Acceptable Product (AP). Conversely, samples yielding inconsistent or ambiguous classification opinions were designated as Uncertain Color (UC). To rigorously challenge the system’s boundary detection capabilities, six UC samples (*n* = 6), specifically within the range of ±1.5, were strategically included in the test batch (*n* = 150). Subsequently, a complete set of samples (*n* = 150), encompassing all possible color grades and the six designated uncertain samples, was randomly fed into the automated sorting machine for classification. The machine’s classification results were then rigorously evaluated against the QC-established ground truth by calculating the confusion matrix and the overall classification accuracy of the system.

## 3. Results and Discussion

### 3.1. Conversion of Spectral Data to Colorimetric Values

**Figure 8:**
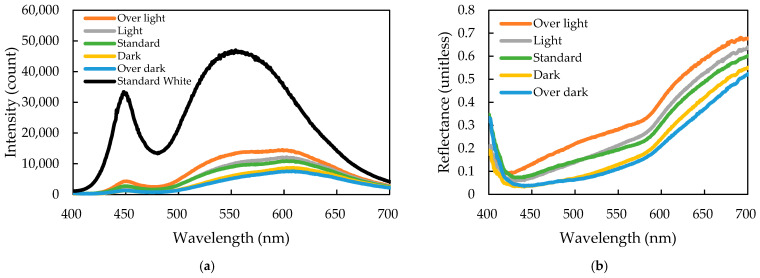
(**a**) Full-wavelength (400–700 nm) light intensity profiles and (**b**) corresponding reflectance spectra of sausages with five smoking levels compared with the standard white reference.

The visible light spectra, using a handheld colorimeter and white standard plaque, revealed two distinct phenomena in 30 smoked sausage samples (Figure 8a). The white standard exhibited significantly higher reflected light intensity, mirroring the LED source’s dual-peak profile (Figure S1b). The five sausage color grades had overall similar spectral shapes but reasonable attenuation in intensity corresponding to the visual order (‘Over light’ to ‘Over dark’). This attenuation was most pronounced in the red-light region (550–700 nm). Such behavior is consistent with optical theory, as darker samples absorb more light. To normalize the data, reflectance was calculated by dividing the sample’s intensity by the white standard measurement’s intensity (Figure 8b). The resulting reflectance curves confirmed that the red-light region was key, providing the sufficient spectral differentiation necessary to clearly separate the five color grades, sequentially ordered from ‘Over light’ (highest reflectance) to ‘Over dark’ (lowest reflectance).

Although low-cost RGB sensors are widely used in consumer imaging, their colorimetric accuracy is strongly influenced by illumination spectra and camera models. In contrast, spectral sensing provides a physically grounded and illumination-aware measurement framework, which is more suitable for reproducible food inspection across different environments [27,28,29].

**Figure 9:**
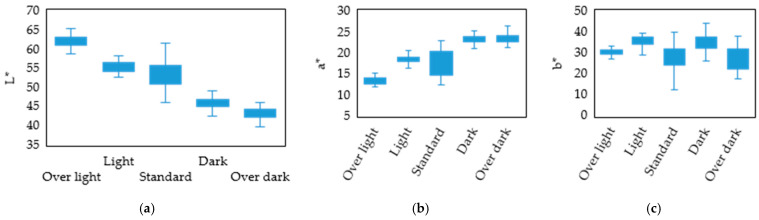
Box plots illustrating the colorimetric distribution of the five smoked sausage color grades: (**a**) L*, (**b**) a*, and (**c**) b*.

The spectral data were subsequently converted into colorimetric values (L*, a*, b*) in the CIE LAB (Figure 9), revealing distinct distribution characteristics for the sausages:

- L* (Lightness): A significant, progressive declining trend was observed from the ‘Over-light’ to the ‘Over-dark’ color grades. The boundaries between groups were well-defined, with a total ΔL* difference of approximately 25, demonstrating excellent discriminative power. This phenomenon is primarily attributed to two factors during high-temperature or prolonged smoke-curing. (1) Water Loss and Structural Change: Surface moisture evaporation leads to shrinkage and structural condensation, altering light scattering properties (increased absorption and decreased reflection), resulting in a darker color [30,31]. (2) Maillard Reaction Products: High temperatures accelerate the reaction between surface amino acids and reducing sugars, generating dark-colored melanoidins [3,32]. Concurrently, phenolic and carbonyl compounds from the smoke deposit and polymerize on the sausage surface [33], forming a dark film that further decreases lightness.
- a* (Redness/Greenness): a* exhibited a slight upward trend as the color deepened. This characteristic was particularly pronounced in the ‘Dark’ and ‘Over-dark’ groups, where a higher a* was generally observed. This feature is helpful for the robust identification of boundary samples. An underlying mechanism of the reaction between nitric oxide (NO) and myoglobin during smoking is the formation of nitrosylmyoglobin. Upon heating, this compound converts into the stable red pigment, nitrosylhemochrome [34,35], which imparts the product’s characteristic smoked red color.
- b* (Yellowness/Blueness): The overall difference in b* was minimal, and the large variance within groups led to significant overlap between color grades. Consequently, the b* component had a limited discriminative effect and was deemed unsuitable as a primary classification basis for smoking intensity.

The observed trends in this study are consistent with existing literature on smoked meat products. To validate the effectiveness of using colorimetry as the basis for classification, a further analysis was conducted on the central tendency and dispersion of the five color grades within the L* and a* axes (Figure 9a,b).

- L* as the Primary Classification Axis: L* is defined as the primary transitional zone between 44 and 61. This zone represents the critical overlap range for the ‘Light,’ ‘Standard,’ and ‘Dark’ grades. The ‘Standard’ group showed a concentrated distribution (Q_1_ = 50.75 and Q_3_ = 55.75), indicating a high probability that samples falling within this specific range are correctly classified as acceptable. However, due to partial overlap, L* alone is insufficient and requires the support of a* for robust determination. Conversely, the ‘Over-light’ (Q_1_ > 61.1) and ‘Over-dark’ (Q_3_ < 44.2) grades were clearly positioned at the extremes of the L* axis, making them suitable as definitive classification boundaries.
- a* as the Secondary Auxiliary Axis: a* effectively discriminates between samples located in the ambiguous L* boundary zone. The data demonstrated that when L* falls between 45 and 48, a sample with a* ≥ 23.9 is highly likely to be categorized as ‘Over dark,’ whereas a sample with a* < 23 tends toward categories such as ‘Dark’ or ‘Standard.’ Furthermore, the a* for both the ‘Dark’ and ‘Over-dark’ grades were predominantly higher than 23.9, serving as an auxiliary criterion for samples exhibiting high redness. Conversely, the ‘Light’ (Q_3_ = 19.03) and ‘Over-light’ (Q_3_ < 14.1) grades exhibited lower a*, providing a secondary feature for their distinction.

In summary, these observations strongly support the two-stage classification logic proposed in this study, with L* as the primary classification criterion and a* as the secondary discriminator in transitional zones. This strategy ensures both statistical robustness and practical operational feasibility for inline sorting.

### 3.2. Validation of Color Grading Threshold Accuracy

**Figure 10:**
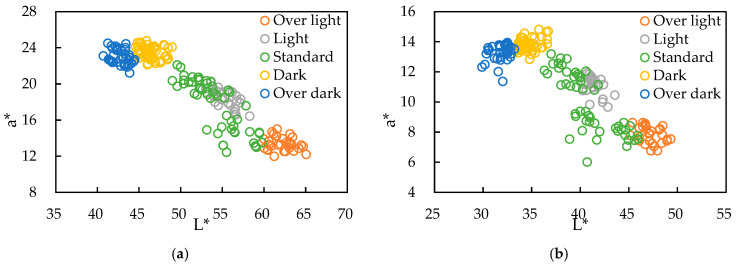
Distribution of the a*-L* two-dimensional scatter plot for the five smoked sausage color grades measured by (**a**) the handheld colorimeter developed in this study and (**b**) a commercial benchtop spectrophotometer (CM-5).

To establish the reliability of the handheld device developed for smoked sausage grading, the two colorimetric values exhibiting significant trends were plotted on a two-dimensional a*-L* scatter diagram (Figure 10a). This plot was then verified against the results obtained from a commercial benchtop spectrophotometer (Figure 10b) to validate the system’s accuracy. Given that the commercial colorimeter did not allow for the export of raw spectral data or internal algorithm settings, the validation focused on comparing the correlation and distributional characteristics of L* and a* (*n* = 30). The comparative analysis of the a*-L* plots (Figure 10) for both the handheld device and the commercial spectrophotometer revealed the following:

- Consistent Color Change Trend and Mechanism: All five color grades (‘Over dark,’ ‘Dark,’ ‘Standard,’ ‘Light,’ and ‘Over light’) demonstrated a consistent trend across both instruments: as the smoking degree increased, L* decreased, and a* increased. This behavior was particularly prominent in the ‘Over-dark,’ ‘Standard,’ and ‘Over-light’ groups.
- Group Overlap: Although the five groups were generally spatially separated, a partial overlap was observed between the ‘Light’ and ‘Standard’ grades on the right side of the distribution. This phenomenon can be attributed to two main factors:
  1. Non-Uniform Smoking Distribution: Traditional smoking ovens often lead to non-uniform heating and smoke distribution depending on the rack position. Samples closer to the heat or smoke source may accumulate higher concentrations of NO and phenols, causing some portions of the batch to shift towards the ‘Standard’ color, while others remain ‘Light.’ Furthermore, the irregular surface of sausages means that variations in the detection angle and reflection characteristics can introduce data dispersion (affecting both human visual and instrumental measurements) [4].
  2. Non-Uniform Surface Composition: The inconsistent distribution of surface moisture and fat significantly impacts the optical properties. Visually, areas where fat has exuded, or surfaces that exhibit a moist sheen, enhance light reflectivity. Conversely, drier areas lead to lower light scattering. This results in the observed non-linear variation and overlapping distribution of L* and a* among the samples [30,31].
- Instrument Resolution Comparison: The handheld colorimeter measured L* ranging from 40 to 65 and a* from 12 to 25. The commercial colorimeter measured L* from 30 to 50 and a* from 6 to 15. Although numerical differences exist due to variations in optical sensing modules and geometric structure, the handheld device demonstrated a wider dynamic range (ΔL* ≈ 25, Δa* ≈ 13) compared to the commercial instrument (ΔL* ≈ 20, Δa* ≈ 9). This suggests that the developed device possesses better resolution for distinguishing subtle variations in smoked color. This finding aligns with comparative studies [5] that show that while smaller spectrometers may exhibit differences in absolute values (and sometimes slightly larger standard deviations than professional instruments), their wide spectral response can provide sufficient, and sometimes more sensitive, discriminative information for specific application scenarios.

**Figure 11:**
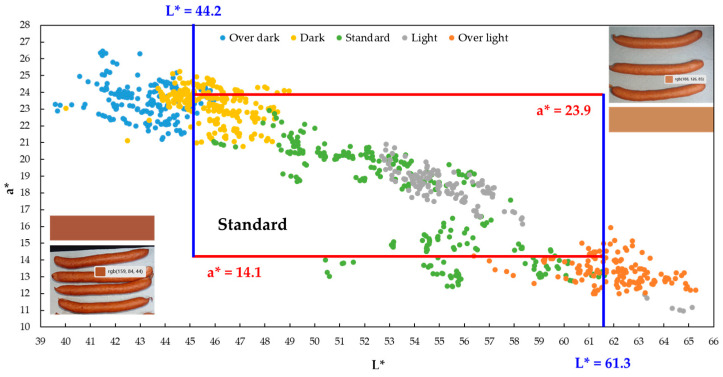
Distribution of a*-L* two-dimensional scatter plot in the color space with defined classification boundaries.

After validating the accuracy of the a*-L* plots with the limited sample set (*n* = 30), the full database (*n* = 180) was utilized to re-examine the color grade distribution and confirm the classification methodology using the expanded a*-L* plot (Figure 11):

- Overall Consistency Trend: The five color grades maintained the common trend where L* decreases as a* increases.
- Increased Overlap: Compared to the smaller *n* = 30 validation set, the degree of overlap between adjacent grades increased significantly. Specifically, the ‘Dark’ and ‘Over-dark’ groups now overlapped, and the ‘Standard’ group showed substantial overlap with both the ‘Light’ and ‘Dark’ groups.
- Specific Group Distribution Shift: Notably, the ‘Light’ group was largely dispersed within the ‘Standard’ group and showed minimal overlap with the ‘Over-light’ group. Instead, the overlap occurred between the ‘Standard’ and ‘Over-light’ groups. This phenomenon is an amplification of the previous validation results, where an increased sample size led to a wider distribution of the ‘Standard’ grade.
- ‘Dark-Brown’ Tendency in the Standard Group: A new phenomenon emerged within the ‘Standard’ group, as samples exhibited a simultaneous decrease in L* but did not show a proportional increase in a*, indicating a shift towards a darker, brownish hue. Potential contributing factors include the following:
  1. Localized Over-Smoking: Excessive smoke concentrations, temperature, or duration in localized areas of the sausage cause phenolic and carbonyl compounds to undergo oxidative polymerization, forming a brown polymeric film on the surface. This film absorbs light in the red and blue regions, preventing a* from rising and resulting in a darker, more brownish color [3,33].
  2. Lack of Surface Sheen: Areas lacking moisture or fat sheen appear visually darker, resulting in a decrease in L*. The effect on a* can be variable [30,31].
  3. Surface Topography: The presence of oil film or wrinkles increases surface roughness, which widens the angle of reflected light. This often causes the single-point sensor to measure lower reflected intensity (decreasing L*) without necessarily changing the overall hue.

The analysis of the expanded database (Figure 11) indicates that the ‘Standard’ group exhibits the largest range of color variation due to localized discrepancies arising from the smoking process. The ‘Over-light’ and ‘Over-dark’ groups represent the extremities of the a* (color) range relative to the ‘Standard’ group’s brightness (L*), while the ‘Light’ and ‘Dark’ groups show a high degree of overlap with the ‘Standard’ group. Consequently, standard acceptance encompasses the entire distribution of the ‘Standard’ group. The redefined and finalized acceptance criteria were set at 45.0 ≤ L* < 61.3 and 14.1 ≤ a* ≤ 23.9.

### 3.3. Calibration of the Optical Inspection Unit in the Inline Automated Sorting Machine

The primary task when calibrating the Optical Inspection Unit (part of the machine shown in Figure 5a) is establishing the data processing methodology for the three sensing modules.

**Figure 12:**
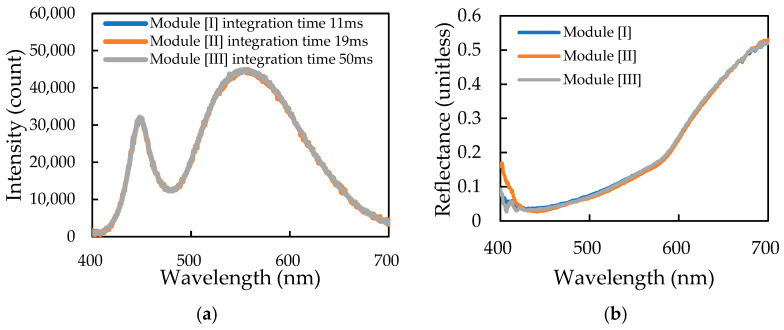
The study of different measurement results from three sensing modules: (**a**) three integration times of three spectrum analyzers obtained by calibration using a PTFE rod; (**b**) reflectance spectra of one sausage in the same position.

(1) Correcting Intensity for Inter-Module Consistency: Calibration was necessary to ensure consistency for the spectral intensity measured by the three modules. Each module measures the same region of the sausage from three different azimuthal positions (spaced 120° apart around the circumference, with each fiber lens/collector positioned 45 mm from the sample). Due to inherent differences in the three modules, such as LED array alignment, incidence angle variation, and spectrometer sensitivity, these measurements must be comparable. A PTFE white rod (Figure 2b), with high uniform surface reflectance, was used as the calibration standard. The objective was to achieve identical sensed spectral intensities across the three modules (Figure 12a) after conversion to a*. This was accomplished by adjusting the spectrometer integration time for each module to 11 ms, 19 ms, and 50 ms, respectively.
(2) Validating Spectral Consistency for Low-Luminosity Samples: The next step verified the spectral consistency of the three modules when challenged with low-luminosity, yet acceptable color grades of smoked sausages. Specifically, we focused on samples from the ‘Standard’ color grade that were close to the ‘Dark’ boundary. The comparison was performed in the central sensing area (the third sensor out of five measurement zones). The results (Figure 12b) showed high spectral overlap in the 420–700 nm range, confirming that the derived a* would be highly consistent and unaffected. Only the short-wavelength region (<420 nm) exhibited greater spectral variation [5]. This suggests that short-wavelength blue light is more susceptible to non-uniform absorption or scattering on the sausage surface, further supporting the decision to exclude b* from the primary classification criteria to avoid similar issues.
(3) Validating Unit Stability and Reproducibility: The final validation confirmed the stability and reproducibility of the entire Optical Inspection Unit (including the conveyor, photoelectric sensors, and the three sensing modules). Challenging ‘Standard’ grade sausages (near the ‘Dark’ boundary) were repeatedly scanned six times, ensuring the same leading end entered the inspection unit. The L*, a*, and b* values analyzed by the three modules for the identical central sensing region were 46.09 ± 0.63, 22.89 ± 0.76, and 35.67 ± 1.09, respectively. The low coefficients of variation (CV) were 1.4%, 3.3%, and 3.1% (Table 3), demonstrating high measurement consistency. This high level of stability confirms that the Optical Inspection Unit can acquire high-repeatability colorimetric data during the automated, high-throughput sensing of large batches of smoked sausages. The scanning process takes approximately 1–2 s per sausage, yielding 15–18 color data points across the surface, which are averaged to represent the overall color of the individual sausage.

### 3.4. Validation of Blind Field Test for the Inline Automated Sorting Machine

To validate the application potential of the developed inline automated sorting machine in an actual production environment, this study conducted three rounds of randomized blind field tests. In each round, 150 smoked sausages were randomly selected, and their color grade was individually determined by the on-site trained QC specialist. The sample sets were designed to cover all five smoking intensity grades. Specifically, the ‘Light,’ ‘Standard,’ and ‘Dark’ grades were aggregated and labeled as an ‘Acceptable Product’ (AP) (Figure S4). The test intentionally included challenging samples: those located near the boundaries of the acceptance zone (threshold L* values of 44.2 ± 1.5 and 61.3 ± 1.5) (Figure 11), and those visually ambiguous samples labeled as ‘Uncertain Color’ (UC) by the trained QC specialists. Consequently, the sorting area utilized four collection bins for ‘Over-light,’ ‘AP,’ ‘Over-dark,’ and ‘UC’ samples. No classification labels were provided during the blind test procedure.

The results demonstrated that the automated sorting machine achieved high classification accuracy across the three test rounds. The accuracies (defined as the same discrimination numbers achieved by the inline automated sorter as those by trained QC specialists for over 150 samples) obtained were 96.0%, 95.3%, and 97.3%, respectively, yielding an average of 96.2% (Table 4). This result confirms the stability and reliability of the developed inline automated colorimeter for high-throughput sorting. Analysis of the misclassified samples by the automated sorter resulted in classifications of ‘Over light’ and ‘Over dark’ for two UC-labeled sausages. Other classification errors primarily involved discrimination differences between the ‘Over-light’ or ‘Over-dark’ groups and the ‘AP’ group (‘Light’ or ‘Dark’). These misclassification errors are analogous to the errors observed in the UC-labeled samples, and only slightly deviate in the classification range. This type of error is mainly attributed to individual sample variability or the inherent limitations of human visual inspection, such as pronounced color non-uniformity on the sausage surface or the difficulty of resolving critical brightness levels near the threshold.

## 4. Conclusions

This study successfully leveraged simple optical components to construct a pivotal sensing module, converting visible light spectral data into the CIE LAB, culminating in the development of both handheld and inline automated colorimeters. The handheld colorimeter utilizes a disposable, curved holder for single-point contact sensing. It decisively establishes that L* and a* are the key colorimetric parameters required to differentiate between the five color grades: ‘Over dark,’ ‘Dark,’ ‘Standard,’ ‘Light,’ and ‘Over light.’ The developed a*-L* database serves as a reliable spot-check tool for on-site quality control personnel.

The Optical Inspection Unit of the automated color sorting machine integrates three sensing modules strategically positioned at different azimuthal locations around the conveyor belt. The system utilizes photoelectric sensors to ensure that each sausage, after excluding its leading and trailing ends, generates five to six distinct measurement zones, yielding 15–18 color data points. These data points are averaged to determine the overall color grade of the entire sausage, which subsequently controls the transport and sorting of the product into designated collection bins. The entire automated inspection and sorting process is completed efficiently within approximately 1–2 s. Robust performance was confirmed through three rounds of randomized blind tests, which yielded an average classification accuracy of 96.2%. The analysis indicated that misclassification errors predominantly occurred near the upper and lower boundaries of the acceptance criteria, suggesting a minimal impact on overall product quality control. This innovative system successfully achieves the standardization, digitalization, and automation of color sorting for flexible, elongated, and curved smoked sausages, thereby realizing the potential for smart manufacturing applications in the processed meat industry.

## Figures and Tables

**Table 1:** Main specifications and functions of a handheld colorimeter’s components.

Components	Specifications	Functions
Ultra Micro Series spectral module	UM2280, OtO Photonic., Taiwan.	Equipped with a built-in color conversion algorithm, capable of directly outputting data in the CIE 1976 LAB color space.
LED	3.2 V, 20 ma, 4000 mcd, LTW-2R3D7, Lite-On Inc., Taiwan.	Provides a light source for detection.
Collimator	NA = 0.22, COL-1-UV, OtO Photonic., Taiwan.	Collimates the optical path.
Optical fiber	NA = 0.22, 200–1100 nm, OF-DS-1000-A, OtO Photonic., Taiwan	Connects the spectrometer and the collimator.
Microcontroller unit (MCU)	ESP32-WROOM-32, Espressif Systems Co., Ltd., China.	The system integrates the operation logic, including operation button control, LED light source driving, display screen update, data storage management, as well as communication with the spectrometer and data reading, and is the main control center of the system.
Real-Time clock module (RTC)	DS1307, Analog Devices, Inc., USA.	Ensure that each measurement data is accompanied by an accurate timestamp.
Monitor	OLED display, resolution 128 × 64 pixels, SSD1306, Solomon Systech Limited, China.	Display L*, a*, and b* along with the classification results.
Lithium polymer battery	10000 mAh, 3.7 V, 1165110, Shenzhen Sunhe Energy Co., Ltd., China.	Power supply.
Single-cell Li-ion battery charger-integrated circuit	TP4056, NanJing Top Power ASIC Corp., Nanjing, China.	Controlling the charging and discharging of lithium batteries (Controls a single 3.7 V lithium battery; provides 5 V power for discharging; charges with 5V1A).
Constant current-integrated circuit	Input 4.2–40 V; output 1.2 V to 37 V; maximum current 1.5 A; LM317, TI, Dallas, TX, USA.	Stable light source current.

**Table 2:** Functional description of major units in the inline automated sorting machine.

Code	Unit	Description
A	Feeding	Responsible for delivering materials into the processing system in a controlled and consistent manner to ensure stable operation.
B	Alignment	Arranges incoming materials into a uniform orientation or orderly sequence, facilitating subsequent quality inspection.
C	Optical Inspection	Equipped with optical and electronic sensors to detect color characteristics of materials during processing.
D	Sorting	Separates and distributes materials into different categories or bins based on predefined criteria for detection.

**Table 3:** Conversion of reflectance spectra into L*, a*, and b* values using three spectrometers with six replicates each in the automated colorimeter. Modules [I]–[III] denote three independent spectrometer modules/channels (installed at different positions in the fixture). All modules were measured at six locations per sausage.

Colorimetric Unit	Number of Measurements	L*	a*	b*
Module [I]	1	46.09	22.41	35.89
	2	46.75	21.74	34.06
	3	46.84	21.71	34.70
	4	46.35	21.96	35.90
	5	46.02	22.13	36.98
	6	45.95	22.31	37.19
Module [II]	1	46.47	23.61	34.71
	2	46.09	23.62	35.09
	3	45.49	23.76	36.19
	4	45.09	23.72	35.03
	5	45.00	23.69	35.25
	6	45.08	23.74	33.46
Module [III]	1	46.17	22.86	35.38
	2	46.87	22.54	35.19
	3	46.90	22.80	36.02
	4	45.54	23.89	36.76
	5	46.39	22.77	37.11
	6	46.53	22.78	37.10
Total	Mean	46.09	22.89	35.67
	Standard Deviation	0.63 (1.4%)	0.76 (3.3%)	1.09 (3.1%)

**Table 4:** Confusion matrix and classification accuracy of the inline automated colorimeter for blind testing (*n* = 150). (The ‘AP’ label contains Dark, Standard, and Light groups; the ‘UC’ label defines the L*-boundaries between acceptable and Over-light/Over-dark sausage samples).

	Inline Automated Sorter	Round 1				Round 2				Round 3			
QC Inspector		Over Light	AP	Over Dark	UC	Over Light	AP	Over Dark	UC	Over Light	AP	Over Dark	UC
Over light		39	1	0	0	38	2	0	0	40	0	0	0
AP		1	58	1	0	1	57	2	0	1	58	1	0
Over dark		0	1	42	1	0	1	42	1	0	0	43	1
UC		1	0	0	5	0	0	0	6	0	0	1	5
Accuracy		96.0%				95.3%				97.3%			

## Data Availability

The original contributions presented in this study are included in the article. Further inquiries can be directed to the corresponding author(s).

## Supplementary Materials

The following supporting information can be downloaded at: https://www.mdpi.com/article/10.3390/s26020678/s1, Figure S1: (a) UM2280 spectrometer used for spectral measurements [23] and (b) emission spectrum of the LED light source (LITE-ON LTW-2R3D7) measured at a 35° emission angle [26]; Figure S2: Electronic circuit design of the handheld colorimeter: (a) lithium battery power supply, (b) TP4056 charging management module, (c) DS1307 real-time clock, (d) SSD1306 display module, (e) LM317 constant-current LED driver, (f) ESP32 logic control.; Figure S3: Voltage variation of the LED light source (LTW-2R3D7) with and without the LM317 constant-current driver: (a) initial power-on without LM317, (b) 10 min after power-on without LM317, (c) initial power-on with LM317, and (d) 10 min after power-on with LM317.; Table S1: Comparison of LED light intensity stability with and without the LM317 constant-current driver.; Figure S4: (a) Sausages were manually classified according to smoking levels and (b) uniform mixing of samples before blind-test validation using the automated colorimeter.
