# Supplementary material for: The Sensor Modules of a Dedicated Automatic Inspection System for Screening Smoked Sausage Coloration
Source: Sensors (Basel). 2026 Jan 20;26(2):678. doi: 10.3390/s26020678 (PMC12846256; doi:10.3390/s26020678)
Supplement: Supplementary file 1 [file sensors-26-00678-s001.zip › sensors-4048951-supplementary.pdf]

Supplementary Materials

S.1 Integration Architecture of IPC and Modbus RTU Communication for the Automated Colorimeter

To realize real-time control and multi-module integration, the developed system utilizes an Industrial Personal Computer (IPC) as the central control unit. The IPC executes spectrometer control, data processing, and external communication logic. The spectrometer is controlled and sampled via the high-speed USB interface on the IPC. Automated control and data exchange, conversely, are managed through the IPC's built-in RS-485 interface using the Modbus RTU communication protocol for reliable bi-directional communication with the on-site Programmable Logic Controller (PLC). The overall design integrates standard optical geometry, constant current illumination, PLC control flow, and multi-point color classification logic, achieving automated capabilities for continuous sample conveyance, real-time measurement, and rapid decision-making.

The IPC serves as the core platform, unifying sensor control, data storage, and production line communication, and is primarily responsible for the following functions:

1. Spectrometer Control and Data Acquisition: The IPC connects to the spectrometer via the USB interface, executing tasks such as start-up, exposure time adjustment, spectral data acquisition, and storage.
2. Communication Synchronization and Data Determination: The IPC's internal RS-485 port implements the Modbus RTU protocol, facilitating the exchange of control commands and the transmission of scanning results with the field PLC.
3. Process Triggering and Feedback: The PLC manages conveyor belt operation and provides photoelectric sensor signals. The IPC periodically reads instruction registers (e.g., D6001) via the Modbus protocol, triggering measurement, executing classification, and subsequently writing the classification results back to designated registers (e.g., D6101).

This architecture provides a highly flexible, modular, and stable real-time communication capability, effectively addressing the stringent demands for data timeliness and precision required in automated industrial applications.

S.2 System-wide Calibration of the Automated Colorimeter

To ensure that the overall automated system operates with consistent, stable, and reproducible measurement conditions in an actual production environment, a comprehensive system-wide calibration procedure must be performed after assembly. This procedure primarily includes two major components: spectrometer luminosity calibration and conveyor belt speed matching, both aimed at unifying the operating conditions of all optomechanical modules to prevent classification errors resulting from individual variances.

1. Spectrometer Luminosity Calibration

The spectrometers utilized in this system feature a 16-bit resolution (maximum value of 65,535). To avoid issues such as saturation, noise interference, and non-linear response, the target reflectance spectral intensity was set to 45,000±5%. The calibration procedure is as follows:

(1) Place the prepared PTFE standard white rod at the center of the measurement view of each of the three optomechanical modules.

(2) Individually adjust the integration time (exposure time) of the three spectrometers until the output luminosity for the standard white sample falls within the predefined target range.

(3) If luminosity remains inconsistent due to mechanical occlusion or variations in light source irradiance, the position or angle of the light sources must be further fine-tuned until the measurement outputs of all three optomechanical modules are uniform.

This calibration step ensures that the system possesses a stable and consistent reflectance intensity under standard conditions, which is essential for subsequent classification accuracy and data comparability.

2. Conveyor Belt Speed Matching Calibration

To synchronize the system with the spectrometer's scanning frequency and exposure time, the conveyor belt speed must be carefully adjusted. The goal is to ensure the sample is sufficiently sampled while traversing the measurement zone, with stable and uniform spacing between sampling points. Based on the set exposure time and triggering frequency, the required dwell time within the measurement zone and the scanning interval were estimated. Multiple trials were conducted in the actual operation to confirm that each moving sample was scanned at least ten times, thereby achieving adequate spatial representativeness and color judgment stability. Following multiple tests and optimizations, the final conveyor belt speed was set to 1.55 m/s. This speed satisfies the required scanning density and synchronizes with the operating rhythm of the downstream sorting mechanism. Through this calibration mechanism, the system achieves stable operation in the industrial application field, characterized by consistent spectral conditions, sufficient scanning coverage, and accurate data-to-physical synchronization.

S.3. Supplementary figures and tables

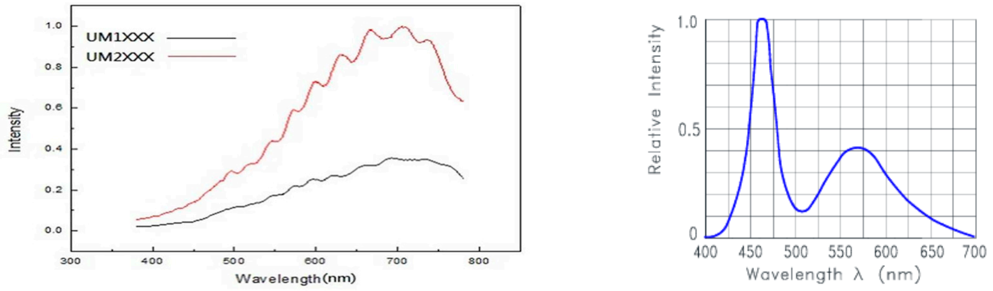

(**a**) (**b**)

Figure S1. (a) UM2280 spectrometer used for spectral measurements[23] and (b) emission spectrum of the LED light source (LITE-ON LTW-2R3D7) measured at a 35° emission angle[26].

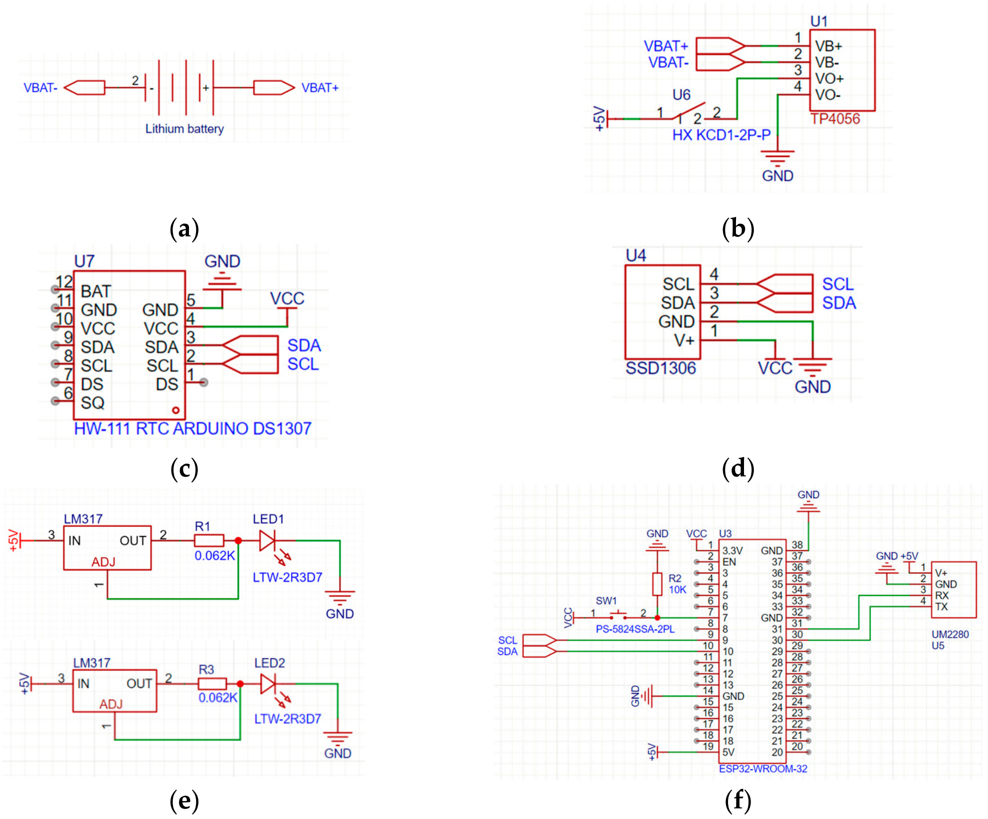

Figure S2. Electronic circuit design of the handheld colorimeter: (a) lithium battery power supply, (b) TP4056 charging management module, (c) DS1307 real-time clock, (d) SSD1306 display module, (e) LM317 constant-current LED driver, (f) ESP32 logic control.

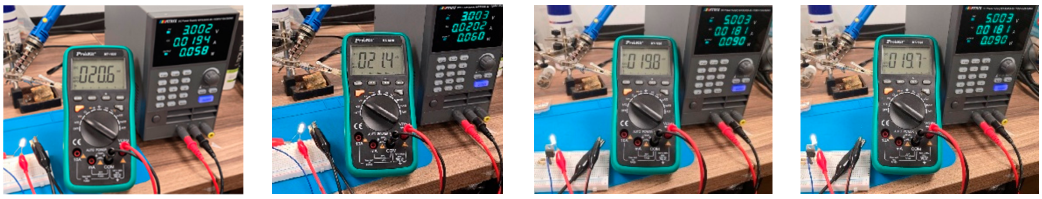

(**a**) (b) (c) (d)

Figure S3. Voltage variation of the LED light source (LTW-2R3D7) with and without the LM317 constant-current driver: (a) initial power-on without LM317, (b) 10 min after power-on without LM317, (c) initial power-on with LM317, and (d) 10 min after power-on with LM317.

Table S1. Comparison of LED light intensity stability with and without the LM317 constant-current driver.

Light intensity (A.U.)	Initial power-on	10 min after power-on	Difference Ratio
without LM317	50337	53114	5.52%
with LM317	48569	48290	0.58%

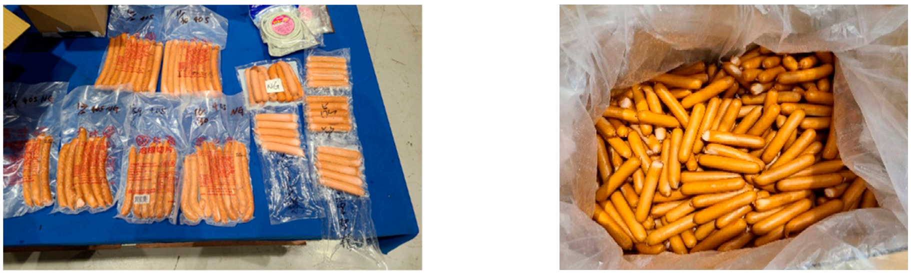

(**a**) (**b**)

Figure S4. (a) Sausages were manually classified according to smoking levels and (b) uniform mixing of samples before blind-test validation using the automated colorimeter.
